# Supplementary material for: Polycomb Repressive Complex 2 promotes atherosclerotic plaque vulnerability
Source: bioRxiv. 2024 Dec 2:2024.12.02.626505. Preprint. [Version 1] doi: 10.1101/2024.12.02.626505 (PMC11656509; doi:10.1101/2024.12.02.626505)

bioRxiv preprint doi: https://doi.org/10.1101/2024.12.02.626505; this version posted December 2, 2024. The copyright holder for this preprint (which was not certified by peer review) is the author/funder, who has granted bioRxiv a license to display the preprint in perpetuity. It is made available under aCC-BY 4.0 International license.

Polycomb Repressive Complex 2 promotes atherosclerotic plaque vulnerability

Divyesh Joshi[1], Raja Chakraborty[1], Tejas Bhogale[1], Jessica Furtado[1,2], Hanqiang Deng[1], James G. Traylor Jr.[3], Anthony Wayne Orr[3], Kathleen A. Martin[1], and Martin A. Schwartz[1,4,5,*]

Supplementary figure legends

Figure S1. RNAseq and ChIPseq analysis. (a) Volcano plot from bulk RNAseq of HUVECs exposed to LSS vs OSS showing shear-regulated genes. (b) X2KWeb analysis to identify common upstream regulators of DEGs from bulk RNAseq analysis of Control si, Pcdhg si and Pcdhg+Klf2/4 triple si HUVECs. Red nodes represent transcription factors while gray nodes represent co-expressed DEGs. The size of the node represents centrality (number of connections with other nodes) in the network. (c) mRNA levels of Ezh2 and Suz12 after Pcdhg depletion. (d) Immunoblot for Ezh2 and Klf4 in Control or Pcdhg si HUVECs under laminar shear stress (LSS) (N=3 independent experiments). Graph: quantitation of Ezh2 and Klf4 normalized to GAPDH loading control. (e) Klf2 promoter region showing peaks for H3K27ac (marking promoter) and H3K27me3 obtained from available ChIPseq analysis on HUVECs (UCSC Genome Browser). (f) H3K27me3 ChIPseq analysis on Control si and Pcdhg si HUVECs showing a significantly higher peak call on Klf2 promoter (N=3 independent experiments). Values are means ± SEM. Statistical analysis used unpaired two-tailed Student's t-test (d).

Figure S2. PRC2 is associated with CVD. (a) De-identified human coronary artery sections from elderly donors stained with H3K27me3 and leukocyte common antigen CD45, comparing regions of plaque (Plaque region) to the regions of the same artery without evident plaque (Control region). Sections were counter stained with DAPI to mark nuclei (N=3 donors). (b-d) Re-analysis of Ezh2, Suz12 and Pcdhg in major plaque cell types including ECs, immune cells and SMCs (b) from published scRNAseq data from deidentified human ASCVD patients comparing regions of plaque (Plaque) to an adjacent plaque-free region of the same artery (Normal)[14]. TNFα and Klf2/4 used as internal controls for plaque ECs and normal ECs respectively (N=3 donors). (c) Feature plot showing inverse correlation between the expression of arterial marker Efnb2 with inflammatory markers Mmp2, Selp and Ccl2 in plaque ECs. (d) Heat map showing Efnb2 levels in Mmp2^{low} (<0.5) vs Mmp2^{high} (>1.5) ECs from plaque.

Figure S3. Expression of Ezh1 and Ezh2 in vascular cells. (a, b) Analysis of mRNA levels of Ezh1 and Ezh2 from human RNA atlas showing a comparison between ECs vs SMCs. Other relevant cell types shown, as indicated. (c) Functional validation of Ezh2 si and Suz12 si confirmed by depletion of PRC2 by Suz12 si or Ezh2 si in HUVECs exposed to OSS and stained for H3K27me3. Scale bar: (c) 20 µm.

Figure S4. Mouse weight and blood lipid analysis. (a-c) Body weights (a), plasma triglycerides (TAGs) and cholesterol content of overnight starved mice treated as indicated (N = 5). Values are means ± SEM. Statistical analysis used one-way ANOVA.

bioRxiv preprint doi: https://doi.org/10.1101/2024.12.02.626505; this version posted December 2, 2024. The copyright holder for this preprint (which was not certified by peer review) is the author/funder, who has granted bioRxiv a license to display the preprint in perpetuity. It is made available under aCC-BY 4.0 International license.

Supplementary Data Table S1. List of primary antibodies.

Antibody	Company, Catalog No.	Dilution for IF	Dilution for immunoblot
H3K27me3	Cell Signaling, 9733	1:800	1:2000
H3K27me3	Abcam, ab6002	1:500	1:2000
Klf4	Abcam, ab215036	1:500	1:4000
H3 total	Cell Signaling, 4499	-	1:2000
Vcam1	Abcam, ab134047	-	1:4000
Klf2	ProSci, 16-624	-	1:2000
GAPDH	Cell Signaling, 5174	-	1:8000
Ezh2	Cell Signaling, 5246	-	1:2000
Suz12	Cell Signaling, 3737	-	1:2000
NICD (Val 1744)	Cell Signaling, 4147	-	1:2000
MEK5	BD, 610956	-	1:2000
ERK5	Sigma Aldrich, E1523	-	1:4000
MMP9	Abcam, ab58803	1:400	-
CD68	ThermoFisher Scientific, 14-0681-82	1:200	-
CD31	R&D Systems, AF3628	1:200	-
SMA	Millipore Sigma, A2547	1:500	-
Erg	Abcam, ab92513	1:800	-
CD45	R&D Systems, AF114	1:200	-

Supplementary Data Table S2. List of siRNAs.

Gene sg/si	Details
Hs Pcdhg si#1	Custom si, Horizon Discovery, CCGCCCAACACGGACTGGCGTTT
Hs Pcdhg si#2	Custom si, Horizon Discovery, CCCAACAACCAGTTTGACACAGA
Hs Klf2 si	Horizon Discovery, L-006928-00-0005
Hs Klf4 si	Horizon Discovery, L-005089-00-0005
Hs Suz12 si	Horizon Discovery, L-006957-00-0005
Hs Ezh2 si	Horizon Discovery, L-004218-00-0005

Supplementary Data Table S3. Hs *Klf2* promoter ChIP-qPCR primers.

	Sequence (5'->3')	Strand	Length	Start	Stop	Tm	GC%
Forward primer	TCCCATCCATCCAGGGTTCT	Plus	20	15	34	59.95	55
Reverse primer	TCAGAGACTCTCAGGGGAGC	Minus	20	124	105	60.03	60
Product length	110						

Fig S1. RNAseq and ChIPseq analysis

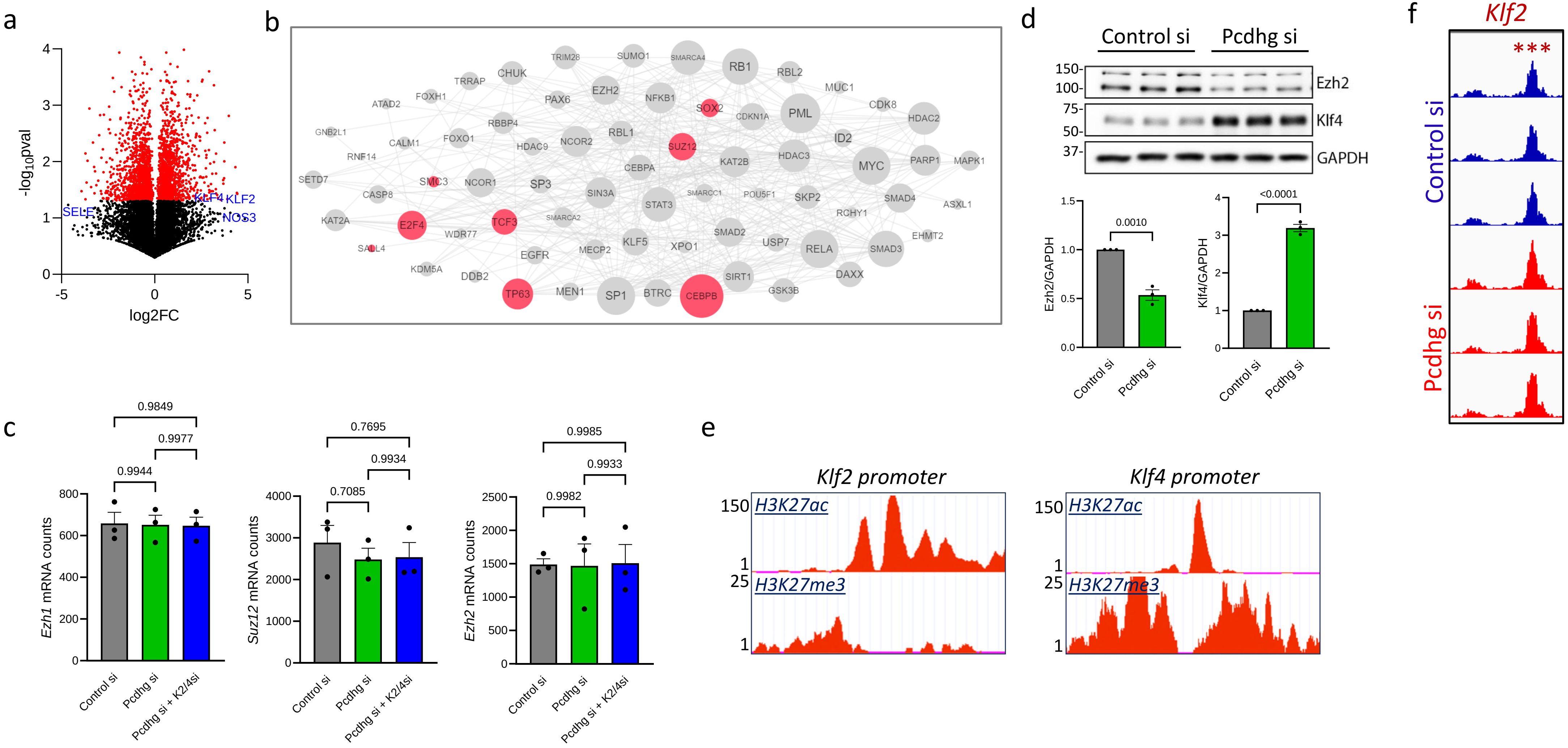

Fig S2. PRC2, like Pcdhg, is associated with CVD

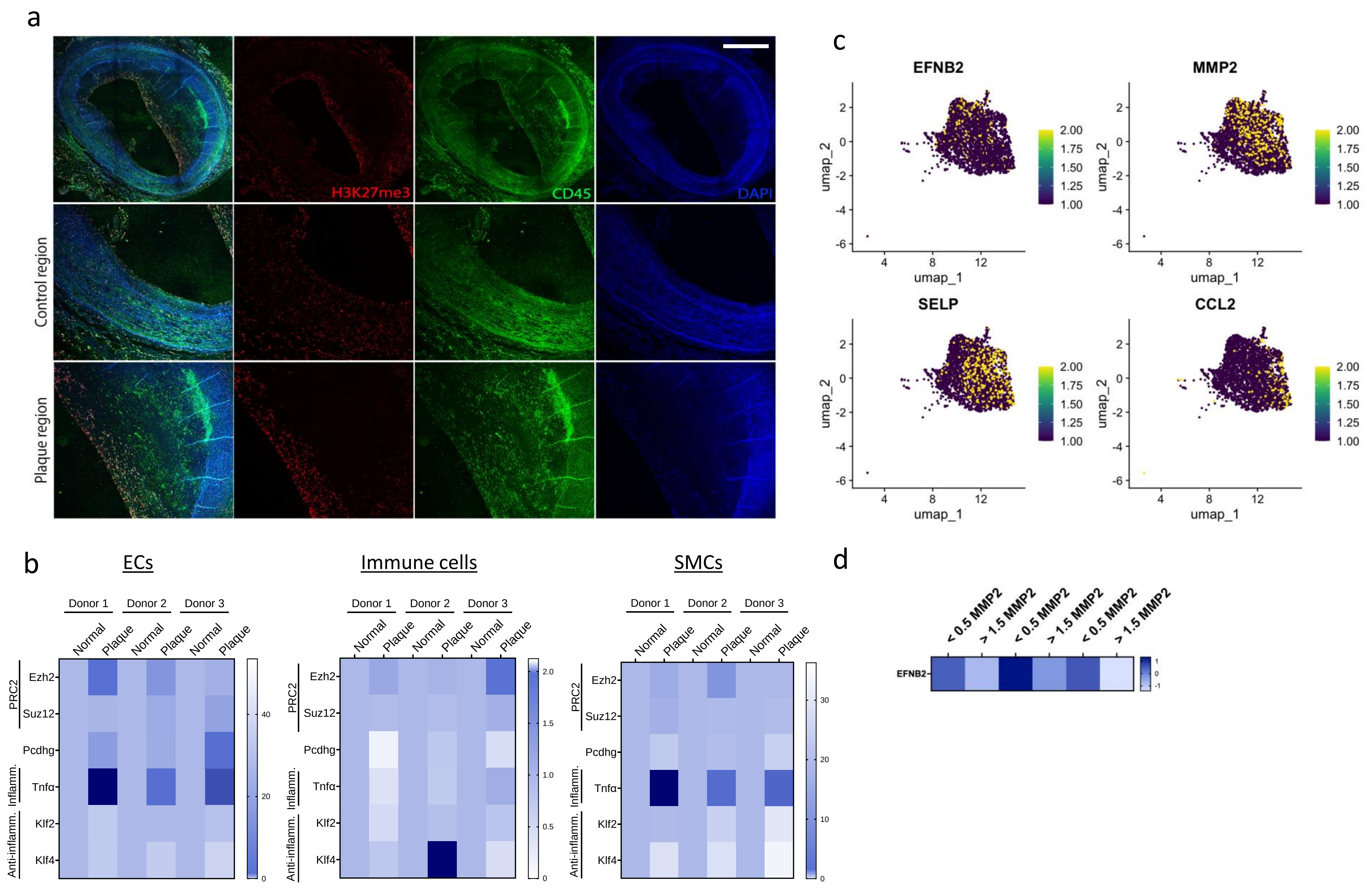

Fig S3. Expression of Ezh1 and Ezh2 in vascular cells

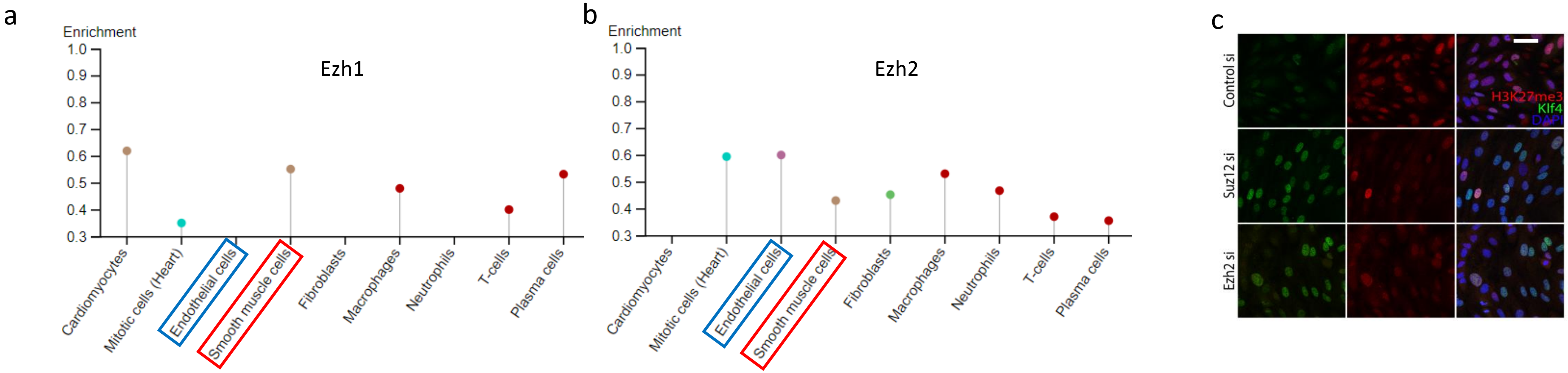

Fig S4. Mouse weight and blood lipid analysis

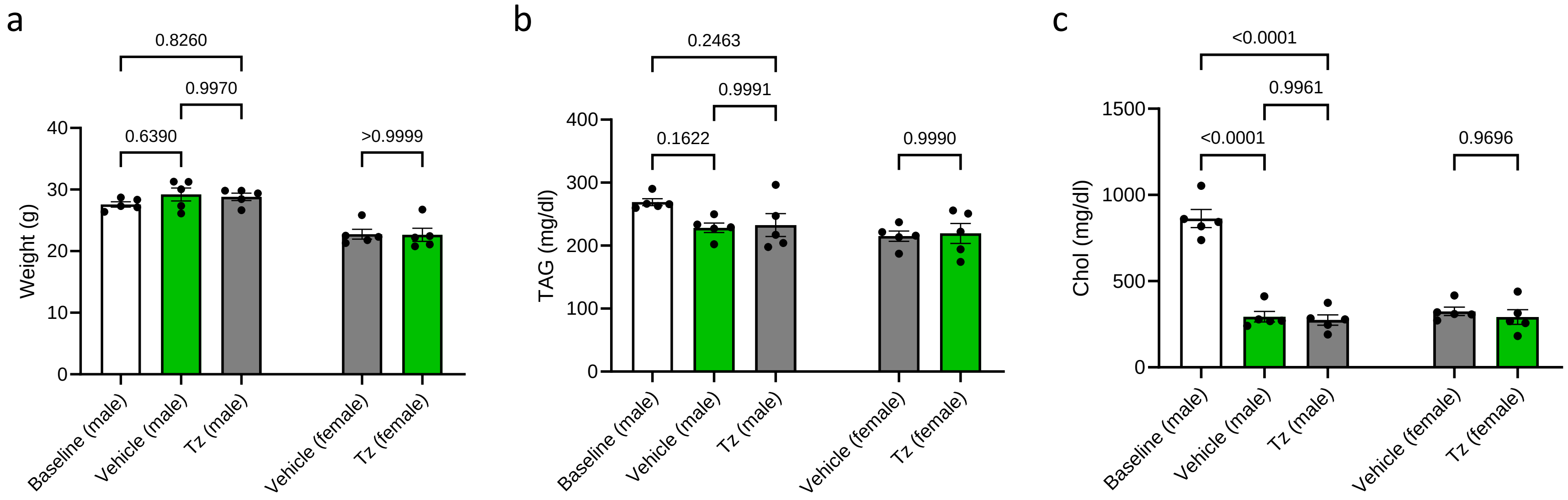

Supplement: 1 [file NIHPP2024.12.02.626505V1-supplement-1.pdf]
